# Psychobiological risk factors for suicidal thoughts and behaviors in adolescence: a consideration of the role of puberty

**DOI:** 10.1038/s41380-021-01171-5

**Published:** 2021-06-11

**Authors:** Tiffany C. Ho, Anthony J. Gifuni, Ian H. Gotlib

**Affiliations:** 1 Department of Psychology, Stanford University, Stanford, CA USA; 2 Department of Psychiatry and Weill Institute for Neuroscience, University of California, San Francisco, San Francisco, CA USA; 3 Psychiatry Department and Douglas Mental Health University Institute, McGill University, Montréal, QC Canada

**Keywords:** Predictive markers, Neuroscience, Psychology

## Abstract

Suicide is the second leading cause of death among adolescents. While clinicians and researchers have begun to recognize the importance of considering multidimensional factors in understanding risk for suicidal thoughts and behaviors (STBs) during this developmental period, the role of puberty has been largely ignored. In this review, we contend that the hormonal events that occur during puberty have significant effects on the organization and development of brain systems implicated in the regulation of social stressors, including amygdala, hippocampus, striatum, medial prefrontal cortex, orbitofrontal cortex, and anterior cingulate cortex. Guided by previous experimental work in adults, we also propose that the influence of pubertal hormones and social stressors on neural systems related to risk for STBs is especially critical to consider in adolescents with a neurobiological sensitivity to hormonal changes. Furthermore, facets of the pubertal transition, such as pubertal timing, warrant deeper investigation and may help us gain a more comprehensive understanding of sex differences in the neurobiological and psychosocial mechanisms underlying adolescent STBs. Ultimately, advancing our understanding of the pubertal processes that contribute to suicide risk will improve early detection and facilitate the development of more effective, sex-specific, psychiatric interventions for adolescents.

## Introduction

Suicide is the second leading cause of death among adolescents ages 10–24 years worldwide [1] and accounts for 17.7% of all deaths in youth ages 15–24 years in the United States [2]. Although suicide has a relatively low prevalence, over the past 10 years suicide rates in the US have increased by 76% in youth ages 15–19 years and, more alarmingly, by 300% in youth ages 10–14 years [3]. Despite the urgency of suicide as a public health problem, research examining predictors of suicidal thoughts and behaviors (STBs) has had limited clinical impact, particularly in adolescents [4, 5]. Adolescents report a higher proportion of suicidal ideation and attempts than do adults, making this a critical developmental period during which to identify early risk factors for STBs [6]. Indeed, the fact that adolescent samples represent only 20% of the literature on predictors of STBs further underscores the need for more research with this age group [5].

While clinicians and researchers recognize that predictors of STBs are multidimensional—including genetic, epigenetic, neurobiological, psychosocial, and environmental factors—the marked increase in the prevalence of STBs (and mental health conditions more generally) during adolescence [7] suggests that this developmental period, in which the confluence of these multidimensional influences contributes to elevated risk, is unique [8, 9]. In this context, we contend that one critical factor is puberty. The onset of puberty initiates a neuroendocrine cascade that shapes the maturation of neural circuits that underlie a range of socioemotional and cognitive functioning, including emotion regulation and impulse control. Impaired emotion regulation and impulse control both significantly contribute to the emergence of mental disorders in the face of environmental risk factors (e.g., life stress) that influence neurodevelopmental trajectories, result in the generation and experience of additional psychosocial stress, and, thus, may represent an important pathway to developing STBs. Other notable aspects of development, such as pubertal timing, and factors that may drive atypical pubertal timing (e.g., exposure to early adversity), are also recognized as important in affecting the development of adolescent-onset psychiatric disorders, including STBs [10]. Indeed, atypical pubertal timing results not only in corresponding changes in neuroendocrine systems but also significant shifts in social identity and perceived status may explain sex differences in the emergence and course of STBs (and related mental health conditions that substantially increase the risk of STBs).

The goal of this review paper is to elucidate how pubertal changes contribute to STBs in adolescence. While we do not regard puberty-related processes as primary determinants of STBs, we posit that exposure to sex hormones during adolescence initiates a period of plasticity in neural circuits that are sensitive to social context (including social stressors that amplify emotion dysregulation and impulse control in ways that increase risk for STBs) and that may be target mechanisms for treatment of STBs, We further contend that these processes are especially critical to consider in adolescents who have existing vulnerabilities, including neurobiological sensitivity to hormonal changes, exposure to adverse psychosocial experiences during early development, and underlying mental disorders. In the following sections, we: (1) present an overarching framework and highlight specific brain circuits involved in social cognition, emotion regulation, and impulse control that are relevant to understanding STBs in adolescence; (2) review basic experimental findings in adults that show the effect of pubertal hormones on behaviors relevant to suicide risk and survey the conflicting correlational literature on hormones with STBs in adolescents; (3) discuss the role of pubertal timing and sex differences in these processes; and (4) advance recommendations for future research in this area.

## Puberty as a driver of neuroendocrine mechanisms relevant for understanding adolescent STBs

Contemporary theories of suicide have been informed primarily by data from adults [11, 12], it is unclear whether these theories—or specific components of associated models—extend to adolescents. Moreover, few theories of suicidality explicitly integrate biological factors, including endocrine or neural factors (with exceptions [12–14]). Here, we posit that adolescent STBs result from a pathological response to stress during a time when the neurobiological systems that regulate stress are recalibrating. There is extensive evidence that STBs in adolescents are often preceded by life stressors, particularly stressors characterized by interpersonal rejection, loss, or conflict [15–17]. Social rejection in particular is a commonly experienced stressor during adolescence due to unstable romantic and peer relationships, and is a potent trigger for negative emotions [18, 19]. In this context, emotion-related impulsivity—the tendency to react impulsively during experiences of heighted affective states [20]—may contribute to adolescent risk for STBs. Indeed, both peer-related stressors [21, 22] and emotion-related impulsivity [23] have been shown separately to predict STBs in adolescents.

We argue here that hormonal changes during puberty alter the development of brain circuits implicated in the regulation of social stressors. The brain is a target organ for all of the pubertal hormones we review, and receptors for these hormones are expressed abundantly in several key structures that comprise brain networks governing social cognition, emotion regulation, and impulse control generally (and social rejection and emotion-related impulsivity specifically) [24, 25], including the amygdala, hippocampus, striatum, anterior cingulate cortex (ACC), medial prefrontal cortex (mPFC), and orbitofrontal cortex (OFC). The amygdala, hippocampus, and ACC comprise a network of regions implicated in detecting social salience; the amygdala and mPFC comprise a key emotion regulatory circuit; the striatum and frontal regions including OFC comprise circuits underlying reward valuation and impulse control. It is important to note that many of these regions already exhibit sexual dimorphism during adolescence, including larger amygdala and hippocampal volumes in boys, greater variance in the hippocampus and striatum in boys than in girls, sex differences in white matter organization of callosal, cerebellar, and long-range association tracts (for a review, see [26]), and sex differences in the developmental trajectories of frontoparietal networks [27]. Moreover, several of these regions have been highlighted in a recent review of neuroimaging markers associated with STBs across the lifespan [28]; however, those authors did not consider the importance of pubertal development in explaining the neuroendocrine basis of the emergence of STBs. We contend that this is a critical factor to consider, given emerging evidence that sex differences in these brain circuits during adolescence appear to be explained, at least in part, by changes in pubertal hormones [29, 30]. Thus, we contend that the social landscape, and most notably environmental stressors, experienced by boys and girls begins to differ during puberty, and that these differences, in conjunction with sex-specific hormonal effects on brain maturation, may explain important sex differences in STBs that emerge by mid-adolescence.

## Pubertal hormones and suicide-relevant thoughts and behaviors

Puberty is composed of two phases: *adrenarche* and *gonadarche*. During adrenarche, which typically begins around ages 7–8 years, the adrenal glands produce increasing levels of the hormones dehydroepiandrosterone (DHEA) and testosterone [31, 32]. Gonadarche is a longer process that typically takes 4–5 years, beginning around ages 9–10 and occurring on average a year earlier in girls than in boys [33]. Gonadarche is triggered by the activation of hypothalamic-pituitary-gonadal (HPG) axis, which leads to rising levels of luteinizing hormone (LH) and follicle-stimulating hormone (FSH). The release of LH and FSH initiates the development of the gonads, which, in turn, leads to increases in sex hormones—specifically, testosterone in boys and estradiol (the predominant estrogen during adolescence) and progesterone in girls—and, by early to mid-adolescence, the development of secondary sex characteristics and other physical changes [32]. Importantly, these hormones cross the blood brain barrier, influence brain development, and affect a wide variety of signaling pathways (e.g., neurotransmitter activity) that underlie mood and cognition [25]. Thus, puberty involves transformation across virtually every psychobiological domain—endocrine, neural, physical, cognitive, and socioemotional—and represents a vulnerable time during which STBs may emerge.

**Fig. 1:**
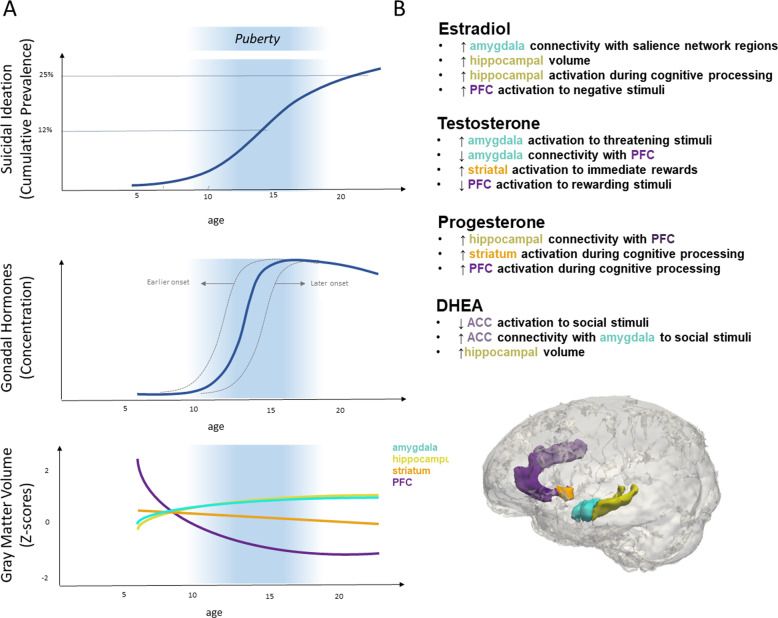
Summary of prevalence rates of suicidal ideation, concentration of sex steroids, and brain volume as a function of age and typical associations between pubertal hormones and brain structures reported the extant literature. **A)** Graphical depictions of prevalence rates of suicidal ideation, concentrations of sex steroids, and brain volume as a function of age. Shaded region indicates puberty. The schematized trajectories of gray matter volume adjusted for total brain volume are based on data reported in [162]. **B)** Summary of typical associations between pubertal hormones and brain structures from both adolescent and adult samples. ACC anterior cingulate cortex, AMYG amygdala, HPC hippocampus, MPFC medial prefrontal cortex, OFC orbitofrontal cortex, STM striatum.

Prevalence rates of STBs begin to increase after age 12 but peak in later adolescence, suggesting that it is not simply the rise in these hormones that accounts for the increase in STBs, given that these endocrine changes begin much earlier than age 12. Instead, the organizational effects of pubertal hormones play a major role in risk for STBs in adolescence. Organizational effects are those that occur during sensitive periods of development that lay the foundation for sex-typical brain and behavioral phenotypes and, thus, have an impact even in the absence of circulating levels of the hormones. In contrast, activational effects facilitate the expression of behaviors under specific contexts but are temporary and occur only when the hormones in question are present [34]. Evidence for organizational effects of hormones during adolescence comes from studies demonstrating that the same developmental processes (e.g., neurogenesis, synaptic pruning, dendritic branching, apoptosis) that occur during the perinatal period as a result of surging levels of gonadal hormones also occur during puberty [35, 36]. These studies suggest that puberty opens a sensitive window for experience-dependent plasticity in neural circuits that underlie higher-order processing of social stimuli, thereby rendering adolescence—a time of increased exposure to social stressors—a vulnerable period for the onset of stress-related mental health problems (Fig. 1). At the same time, it is possible that ongoing fluctuations in hormone levels are also related to STB risk through their activational effects on neurotransmitter systems, particularly in the context of responding to social stressors, and that these effects are more relevant for adolescents with a neurobiological sensitivity to hormonal changes.

Below, we review studies investigating ovarian (estradiol and progesterone), testicular (testosterone), and adrenal (DHEA) hormones in relation to STBs and, importantly, their impact on the structural development and function of specific brain circuits implicated in social cognition, emotion regulation, and impulse control. A summary of this section is outlined in Table 1 and Fig. 1B. Before reviewing the correlational literature in adolescents, within each respective section, we first review basic experimental studies that provide insight concerning the effects of estradiol, progesterone, testosterone, and DHEA on human behaviors relevant to suicide risk in adults in order to provide a more reliable context for the actions of these hormones.

**Table 1:** Summary of studies examining gonadal and adrenal hormones in relation to suicide attempts and suicidal ideation.

Publication	Sample size and characteristics	Age (years)	Sex	Psychiatric condition	Suicide-related outcome	Study design and methods	Findings	Additional notes
Afzali et al. 2012	81 suicide attempters	Mean = 23.63 SD = 8.41 Range = 15–55	F	Assorted (25 Past mental disorder, 22 Previous suicide attempt)	History of Suicide Attempts	Structured interview over 6 months after attempt	Suicide attempts were not associated with menstrual cycle phase.	Patients with irregular menstrual cycles were excluded.
Baca-Garcia et al. 2010^a^	281 suicide attempters 176 healthy controls	Mean = 30.8 SD = 8.8 Range = 18–92	F	Assorted (229 Mood disorder, 229 SUD, 275 Previous psychiatric treatment)	Recent Suicide Attempts and Recent Suicidal Ideation	Blood sample within 24 h of attempt: estradiol, progesterone, LH, FSH	Suicide attempts were was more likely during the follicular phase. Suicide intent severity was elevated during low-estrogen/low-progesterone states (pre-menstrual phase, amenorreha, menopause)	
Butterfield et al. 2005	130 inpatients	Mean = 49.4 SD = 8.13	M	PTSD	Recent History of Suicide Attempts (past 6 months) and Suicidal Ideation	Blood: DHEA, androstenedione, testosterone, estradiol	Suicide attempters had higher DHEA than nonattempters	
Cayköylü et al. 2004^a^	52 suicide attempters 50 healthy controls	Mean = 26.51 SD = 7.82 Range = Not Reported	F	Assorted (8 PMDD, 1 SCZ, 2 MDD, 1 OCD)	Recent Suicide Attempts	Blood sample within 12 h of attempt: estradiol, progesterone Menstrual status determined with self-report.	Suicide attempts were more frequent during the follicular phase. Estradiol and progesterone levels were not different in suicide attempters compared to healthy controls.	Patients attempting suicide with OD or admitted to the ICU were excluded.
Chatzittofis et al. 2013	28 suicide attempters (10 female, 18 male) 19 healthy controls (7 female, 12 male)	SA: Mean = 44 SD = 14.6 Range = 26–66 HC: Mean = 30 SD = Not Reported range = 23–48	Both	Assorted (14 Mood disorder, 4 Anxiety Disorder, 9 SUD, 19 PD)	History of Suicide Attempts	CSF: DHEA-S, DHEA, cortisol, and 5-HIAA	In males, suicide attempters had higher CSF DHEA-S levels compared to healthy controls. In females, no significant differences.	Exposure to early adversity (e.g., interpersonal violence) correlated negatively with cortisol/DHEA-S ratio
Dogra et al. 2007^a^	217 suicide decedents 237 non-suicide decedents	45% of suicide dececents ages 21–30 Bimodal distribution in the non-suicide decedents: 23% ages 20–25, 23% ages 30–35 Range = 11–45	F	Not Reported	Suicide Death	Autopsy Menstrual status determined by visual examination of the uterine cavity	54.46% of non-pregnant women who died by suicide were menstruating versus 6.75% in the non-suicide decedent group	
Fouriestié et al. 1986^a^	108 suicide attempters	Mean = 25.3 SD = 4.0 Range = Not Reported	F	Assorted (9 with previous psychiatric admission, 15 treated with neuroleptic and/or antidepressant medication, 9 treated with anxiolytics, 15 with previous suicide attempts)	Recent Suicide Attempts	Blood sample within 12 h of attempt: estradiol and progesterone	Suicide attempts were more likely to happen during phases with low estradiol, during the first week of the menstrual cycle (42%) and after the fourth week (12%). Frequency of suicide attempts did not vary significantly during the menstrual cycle in OC users.	Patients admitted to the ICU were excluded.
Gustavsson et al. 2003	43 suicide attempters	Mean = 38.0 SD = 12.0 Range = Not Reported	M	Assorted (14 SUD, 9 DDNOS, 10 MDD, 4 Dysthymia, 9 Adjustment disorder, 4 Anxiety disorder, 2 Psychosis)	Recent Suicide Attempts	CSF in days (5–57 days, mean = 16) following suicide attempt: testosterone	Suicide attempters with depressive disorders showed higher CSF testosterone than those with other psychiatric diagnoses.	CSF testosterone positively correlated with irritability and negatively correlated with social desirability.
Papadopoulou et al. 2018^a^	70 suicide attempters	Mean = 35.5 SD = 8.9 Range = 18–52	F	Assorted (28 MDD, 13 BD, 14 Psychosis, 15 PD or adjustment disorder)	Recent Suicide Attempts	Blood sample within 72 h of suicide attempt or within 48 h after transfer to the ICU: progesterone, LH, FSH Menstrual status determined with progesterone levels, LH and FSH were used to rule out menopausal status	Suicide attempts were more frequent in the last 4 days of days of luteal phase and during the 4 days of menses.	No effect of menstrual status on lethality (violent vs non-violent mode of attempt) or psychiatric diagnosis.
Markianos et al. 2009^a^	15 suicide attempters (intentional jumps) 18 accident victims (falling from a height) 40 healthy controls	SA: Mean = 39.9, SD = 14.3, Range = 22–62 Non-SA: Mean = 37.6 SD = 15.2 Range = 20–66 HC: Mean = 31.6 SD = 9.0 Range = 25–59	M	Assorted (10 SCZ, 5 MDD)	History of Suicide Attempts	Blood: testosterone, LH, FSH	Suicide attempters had lower levels of testosterone (trending, p = 0.065) and LH compared to accident victims. Both suicide attempters and accident victims had lower levels of testosterone and LH compared to HC.	
Martin et al. 1997^b^	81 female and 79 male adolescents	Mean = 16.0 SD = 1.0 Range = 15–19	Both	Not Reported	History of Suicide Attempts and Suicidal Ideation	Blood: progesterone	In males, progesterone was higher in those with past suicide attempts and with suicide ideation. In females, progesterone levels negatively correlated with past suicide attempts and disclosed suicide ideation	SUD excluded from analyses.
Roland et al. 1986	39 suicide decedents 48 non-suicide (sudden death) decedents	SA: Mean = 39.1 SD = 18.3 Range = 15–76 HC: Mean = 51.5 SD = 13.8 Range = 12–79	M	Not Reported	Suicide Death	Autopsy Blood: testosterone	Suicide decedents showed higher levels of testosterone compared to non-suicide decedents.	
Sher et al. 2012	67 patients with bipolar disorders and at least one past suicide attempt (51 female, 16 male)	Mean = 34.5 SD = 9.9 Range = 18–75	Both	Bipolar Disorder	History of Suicide Attempts	Blood: testosterone	Testosterone levels positively correlated with the number of past suicide attempts, while controlling for sex.	Testosterone levels were also positively correlated with number of manic episodes, while controlling for sex.
Sher et al. 2014	51 patients with bipolar disorder and at least one past suicide attempt	Mean = 33.2 SD = 9.6	F	Bipolar Disorder	History of and Prospective Suicide Attempts (prospective follow-up for up to 2.5 years)	Blood: testosterone	At baseline, testosterone levels positively correlated with the number of suicide attempts and past major depressive episodes. Higher testosterone levels predicted suicide attempts in the follow-up period.	
Sher et al. 2018^a^	17 combat veterans with post-deployment suicide attempt (0 female, 17 male) and 17 non-suicidal combat veterans (2 female, 15 male)	SA: Mean = 37.5 SD = 11.6 Non-SA: Mean = 35.7 SD = 10.8	Both	PTSD	History of Suicides Attempt and Suicidal Ideation	Blood: DHEA, DHEA-S	Suicide attempters had lower levels of DHEA and DHEA-S compared with nonattempters. Suicidal ideation negatively correlated with DHEA and DHEA-S levels across all participants. Suicidal ideation negatively correlated with DHEA-S levels in nonattempters.	DHEA/DHEA-S ratios positively correlate with adolescent and adult aggresion scores in suicide attempters.
Stefansson et al. 2016	28 suicide attempters (10 female, 18 male) 19 healthy controls (7 female, 12 male)	SA: Mean = 44.0 SD = 14.6 Range = 23–66 HC: Mean = 30.0 SD = Not Reported Range = 23–48	Both	Assorted (MDD, PTSD, SUD)	Recent Suicide Attempts and Prospective Suicide Death (prospective 21-year follow-up)	CSF and blood in days (mean = 8.6, range = 2–17 days) following suicide attempt: testosterone, cortisol	In males, CSF and blood testosterone levels were higher in suicide attempters compared to healthy controls. In females, no differences.	In males, CSF testosterone/cortisol ratio positively correlated with impulsivity and aggressiveness in the suicide attempters. No differences associated with MDD, PD, or SUD
Tripodianakis et al. 2006	80 suicide attempters (29 with schizophrenia) 56 healthy controls 29 nonattempters with schizophrenia	SA: Mean = 34.4 SD = 12.6 HC: Mean = 35.3 SD = 8.7	M	Schizophrenia	History of Suicide Attempts	Blood: testosterone, LH, FSH	Suicide attempters had lower blood testosterone compared to healthy controls. Attempters with schizophrenia had lower levels of testosterone compared to nonattempters with schizophrenia.	Attempters who used a violent method had lower testosterone levels than non-violent attempters.
Zhang et al. 2015	245 suicide attempters (172 female, 73 male) 245 healthy controls (172 female, 73 male)	SA: Mean = 42.9 SD = Not Reported Range = 16–50 HC: Mean = 37 SD = Not Reported Range = 14–53	Both	Not Reported	History of Suicide Attempts	Blood: testosterone	In males, testosterone was higher in male suicide attempters compared to healthy controls. In females, no significant differences.	

*F* female, *FH* follicular hormone, *DDNOS* depressive disorder not otherwise specified, *ICU* intensive care unit, *LH* lutenizing hormone, *M* male, *MDD* major depressive disorder, *OC* oral contraceptive, *OD* overdose, *OCD* obsessive-compulsive disorder, *PTSD* post-traumatic stress disorder, *SA* suicide attempt, *SCZ* Schizophrenia, *SD* standard deviation, *SUD* substance use disorder.

^a^Low-quality study due to limited sample size and/or limitations in study design (e.g., single-timepoint cross-sectional associations between ovarian hormones and suicidal thoughts and behaviors).

^b^Adolescent sample.

Finally, because ovarian hormones have been investigated almost exclusively in females in this context and, similarly, androgen hormones overwhelmingly in males, our review of the association of these hormones with brain and behavioral outcomes inherently raises issues of sex differences or sex-specific effects in these processes; we discuss these issues in more detail in a later section (D. Consideration of sex differences).

### Ovarian hormones (estradiol and progesterone)

Estradiol, progesterone, and their neurosteroid metabolites all increase and begin to fluctuate cyclically in girls during puberty [34]. Consequently, the vast majority of research in this area has focused on females (for reviews, see [14, 37]). To provide a context for understanding the effects of estradiol and progesterone administration on behaviors relevant to suicide risk, we will first briefly review studies that controlled hormone conditions in women with affective symptoms and premenstrual dysphoric disorder (PMDD), for whom suicide risk is elevated compared to the general population [38, 39].

Decades of research have shown that there are no differences between women with and without PMDD in ovarian hormone levels or related neurosteroids (e.g., allopregnanolone) but that suppressing ovarian hormones reduces or eliminates symptoms of PMDD (for a review, see [40]). A recent study that controlled for ovarian hormone secretion and exposure in women with PMDD has helped to clarify these two seemingly opposing findings [41]. In that study, women with PMDD who responded to a gonadotropin-releasing hormone agonist treatment were given placebo for one month before being administered continuous estradiol/progesterone for three months; researchers found that changes from low to high levels of ovarian hormones, but not absolute levels of ovarian hormones, were associated with increases in negative affect [41]. Together, these data suggest that neurobiological sensitivity to hormone *changes* is an important factor that may explain certain clinical phenomena, such as PMDD and suicide risk. Indeed, a recent review has covered this topic extensively in adult women, demonstrating that cyclical hormone changes may play an important role in “acute risk for daily suicidal ideation, planning, and intent” in individuals with sensitivity to hormone changes [14]. We have extended this theoretical model to adolescents, proposing that in those who possess a neurobiological sensitivity to hormonal changes, the normative fluctuations during this transitional period, coupled with adolescent-typical experiences of greater exposure to life stressors, may exacerbate the effects of these processes on emotion regulatory and impulse control circuits. Indeed, results from studies that have investigated suicide risk in adult women across the menstrual cycle have been inconsistent. These studies have typically assessed levels of estradiol and progesterone during different phases of the menstrual cycle [42–44], and in women with low levels of estradiol and progesterone (e.g., amenorrhea or menopause [45]). While some investigators have reported that a higher risk of suicide attempts and more severe suicidal thoughts and intentions are associated with relatively low or declining levels of estradiol and progesterone (i.e., during the early follicular/menstrual or pre-menstrual phases), other researchers have not found differences in estradiol or progesterone between depressed women with and without STBs [46] or effects of the menstrual cycle on suicide attempts in women with PMDD [39].

The fact that there has been no evidence of differences in ovarian hormone levels or in cyclical changes in female adolescents with and without STBs is consistent with the previously aforementioned studies in adult women with reproductive mood disorders where the effect of menstrual cycle and/or ovarian hormone levels on STBs in adult women is absent. One interpretation is that it is the neurobiological sensitivity to *changes* in estradiol and progesterone—rather than between-person differences in levels of these hormones—that represents a key trait-like source of variance for understanding suicide risk in the context of puberty, a time when the stability of these hormone dynamics is still in flux [47]. Intensive (e.g., daily samples) longitudinal studies conducted in adult women have also provided little evidence that hormone levels at a single point in time are correlated with levels within the same cycle and also for subsequent cycles, particularly for estradiol [48, 49]. It is also important to note that both between-person and within-person variability of ovarian hormone levels are affected by multiple confounds that investigators ought to consider, including, but not limited, to cycle length [50], diurnal effects [51], cycle phase [48, 49], effects of study participation [48], anovulation [52], culture and/or diet [53], and personal and family medical history (e.g., polycystic ovary syndrome, breast cancer [48, 54]). Nevertheless, dense-sampling studies have demonstrated robust between-person effects of menstrual phase, such that progesterone is reliably higher in the luteal phase relative to the follicular phase and, albeit to a lesser extent, estradiol is higher in the follicular phase relative to the luteal phase [48, 49]. We note in Table 1 which studies employ non-experimental cross-sectional designs and that attempt to relate ovarian hormones without consideration of these relevant factors (e.g., cycle phase).

Given these limitations in measuring ovarian hormones, it is not surprising that little is known about the effects of estradiol and progesterone on the human brain in general, much less on the circuits we have hypothesized are implicated in STB risk. Nonetheless, there is evidence that estrogen receptors are expressed strongly in brain regions involved in social cognition broadly and social rejection specifically, including the amygdala, hippocampus, ACC, and vmPFC [55–58]. Several studies have also found that women with comparably higher levels of estradiol (endogenous or synthetic) show greater amygdala-based resting-state functional connectivity and activation in ACC and vmPFC, which are key regions involved in processing salient information [59–61]. Similarly, longitudinal studies with naturally cycling women have documented larger hippocampal gray matter volumes during periods of relatively high estradiol (late follicular/preovulatory phases) than of relatively low estradiol early follicular/premenstrual phases [62, 63].

There is also evidence from functional magnetic resonance imaging (fMRI) studies wherein groups of women during different phases of their menstrual cycle are compared that show that relatively higher levels of estradiol (pre-ovulatory phase) are associated with greater activation of the hippocampus during both cognitive tasks [64] and affective stress tests [65], and with stronger functional connectivity of the hippocampus with brain regions involved in processing salient information [63]. Similarly, higher levels of progesterone (luteal phase) have also been found to be associated with activation of the striatum and PFC during cognitive processing [64]. Finally, results from fMRI studies utilizing dense-sampling designs of naturally cycling women have reported intriguing, albeit inconsistent, results. One study found no effects of estradiol across the menstrual cycle on intrinsic connectivity patterns [66], whereas another found that higher levels of estradiol drove stronger subsequent connectivity within attention networks (specifically among brain circuits that are implicated in internally focused attentional states and externally focused attentional states) [67]. In contrast, one study found that progesterone mediated patterns of positive functional connectivity between the hippocampus and PFC [66] whereas another study found that higher levels of progesterone was associated with lower connectivity across all networks [67]. It is clear that additional research is needed to clarify the magnitude and directionality of the effects of estradiol and progesterone on patterns of brain connectivity, particularly in adolescents, and their effects on longer-term neurodevelopmental trajectories. It is also critical that researchers work to characterize the extent to which these hormones effect brain circuits specific to STBs, or alternatively, whether they are implicated in mental illness risk more broadly. Nevertheless, the studies we have reviewed provide initial evidence that brain circuits that support the regulation of affectively salient stimuli are sensitive to the effects of estradiol and progesterone; importantly, these same circuits have also been shown to be affected in adults and adolescents with STBs [28, 68].

Additional research is also needed to elucidate the precise neural mechanisms by which estradiol may impact the development of brain circuits, including its effects on neurotransmitters. Estradiol has also been found to alter serotonin transmission, binding, and metabolism by increasing the production of tryptophan and inhibiting the expression of the serotonin reuptake transporter gene (for a review, see [69]). Serotoninergic abnormalities in the number of serotonergic neurons and in serotonin transportation, receptor binding, and levels have all been found in victims of suicide [70, 71] (for a review, see [72]). Serotonin has been shown to be associated with cortisol reactivity to stressors [73] and positively associated with greater 5-HT_1A_ receptor binding—which could contribute to lower serotonin signaling by inhibiting further serotonin release into the synapse—in depressed patients who died by suicide versus both depressed and psychiatrically healthy individuals who did not die by suicide [74]. Similarly, allopregnanolone, a progesterone metabolite, binds to GABA_A_ receptors, which mediates the majority of inhibitory signaling in the brain [75] and plays an important role in downregulating the HPA-axis in response to acute stressors [76]. Several studies have found that stress exposure alters the availability of GABA_A_ receptors as well as their composition and sensitivity to neurosteroid regulation, which, in turn, can influence subsequent responses to stress [77–79]. Emerging data has also implicated GABA dysfunction in STBs, whereby depressed patients who died by suicide showed a higher expression of genes that encode for proteins involved in GABAergic synaptic transmission in the ACC and a lower expression of these genes in the dorsolateral PFC than did both depressed and psychiatrically healthy individuals who did not die by suicide [80]. Future work is needed to explicitly investigate the extent to which patterns of structural or functional connectivity of circuits involving the amygdala, hippocampus, ACC, and PFC that are developing in response to puberty-related changes in estradiol and progesterone (and related neurosteroids) exhibit corresponding changes in neurotransmitter systems that support socioemotional processes relevant to STB risk.

### Testosterone

Unlike ovarian hormones, which have been studied primarily in female adolescents, investigators have found in both sexes that levels of androgens are associated with appetitive behaviors, aggression, competition, and other related social behaviors that are relevant to STB risk [81, 82]. In female adolescents, the sex steroid with the most androgenic activity is DHEA, which is produced by the adrenal cortex (discussed separately in the following section); in males adolescents, it is testosterone [31, 32].

In contrast to the data published thus far on ovarian hormones, there is evidence of strong within-person reliability and stability in testosterone levels in both men and women. Indeed, some researchers contend that levels of testosterone are trait-like [83]. Perhaps not surprising, testosterone has been linked more strongly with STB risk, and specifically with suicide attempts, than has any other pubertal hormone (see Table 1). However, the empirical findings thus far have been mixed (for opposing reviews, see [84, 85]). Several researchers have theorized that testosterone is linked to suicidal behaviors through its modulation of emotion-related impulsivity and impulsive aggression, which are considered to be among the most robust predisposing traits to suicidal behaviors in youth [86]. In drawing from the literature of studies in which testosterone is administered and behaviors are subsequently assessed, as well as studies that link changes in social interactions with changes in endogenous testosterone, there appear to be reliable effects of testosterone on socially motivated behaviors, including exerting dominance and displays of aggression (either physically or non-physically) and other social status seeking behaviors (for a review, see [87]). While testosterone exert complex effects on interpersonal behavior, longitudinal studies show that the puberty-related increases in testosterone are not accompanied by a concurrent rise in aggressive behaviors [83, 88, 89]. Additional studies in animals as well as in humans [90] suggest that testosterone levels correlate more closely with social dominance, rather than aggressive behaviors. Hence, testosterone may be an important moderator of the behavioral response to events associated with loss of social status [91], which are known precipitants of STBs. In the context of adolescent STB risk, it may very well be that testosterone is a driver of heightened sensitivity to social context, which can lead to significant emotion dysregulation and impulse control and, in turn, elevated STB risk.

Consistent with these points is evidence that elevated testosterone has also been found in male adults to be associated with psychological states and individual traits associated with suicide risk, including depression severity, irritability, and impulsive aggression [92, 93]. Researchers have found higher levels of testosterone in both male and female suicide attempters than in their same-sex non-attempting counterparts [94], and in post-mortem samples of suicide completers compared to individuals who died from other causes [95]. In a recent study, male, but not female, young adults who attempted suicide had higher levels of testosterone than did age- and sex-matched healthy volunteers [92]. Thus, there studies do not preclude the possibility that testosterone levels are higher in individuals with a mental disorder than they are in healthy persons. One study found in women with bipolar disorder with a history of suicide attempt that higher testosterone levels predicted subsequent suicide attempts [96]. Interestingly, in another sample of men and women with bipolar disorder, higher levels of testosterone were associated with number of suicide attempts only after controlling for sex [97]. In contrast, other studies have found lower levels of testosterone both in male suicide attempters relative to psychiatric healthy controls [98] and in patients who were hospitalized due to accidents [99]. One possible discrepancy for these findings involves the measurement of testosterone in plasma versus cerebrospinal fluid; studies assaying plasma testosterone have typically reported inverse associations with suicide attempts. Nevertheless, it is clear that adequate clinical controls (attempters or completers relative to nonattempters who otherwise have similar clinical histories) must be included in research in order to clarify the results of these studies.

As is the case with research on ovarian hormones and STBs, no studies have examined the psychobiological mechanisms and specific brain circuits through which testosterone may drive risk for STBs in either adolescents or adults. Testosterone influences many of the same neurotransmitters as does estradiol [25]; moreover, testosterone can be converted into estradiol via aromization [100], adding additional complexity in these processes should be addressed in future work. That said, one pathway through which testosterone may affect mood and cognition is through altering dopaminergic neurotransmission: several studies have found evidence of differences between suicide attempters and completers in dopamine receptor density, transporter binding capacity, and metabolism [101–103], primarily in the striatum. In fact, testosterone receptors are widely expressed in dopaminergic neurons of the striatum and portions of the PFC, including vmPFC and OFC [104]. Research with both macaques and humans has documented changes in the mechanisms that underlie dopamine signaling during adolescence, which may be explained, in part, by effects of testosterone (and estrogen; for reviews, see [105, 106]). In particular, testosterone has been shown to increase basal dopamine levels and decrease the number of enzymes that break down dopamine in the striatum and PFC [105]. Consequently, it is possible that adolescent-typical increases in testosterone during puberty, especially in boys, contribute to elevated STB risk through enhanced dopaminergic transmission. This is consistent with previous reports that, compared to depressed patients who did not die by suicide, depressed patients who die by suicide have lower growth hormone responses to apomorphine [102, 107], which is an indication of higher dopamine transporter binding [108]. Nonetheless, it is important to note that some studies provide evidence of lower dopamine (measured by lower levels of homovanillic acid and total dopamine in urine) in patients who attempted suicide versus those who did not, see [103], or of no significant associations between dopamine levels/receptor binding and STBs [109].

With respect to brain development, higher levels of pubertal testosterone have been associated with white matter organization in tracts implicated in social cognition and emotion regulation, including the uncinate fasciculus (which connects the amygdala and vmPFC) and corpus callosum in both boys [110, 111] and girls [29, 112]. Several researchers have documented other effects of testosterone on adolescent and young adult brain and behavior. Human neuroimaging studies using fMRI have shown that in healthy young men, activation in the amygdala—a brain structure that is rich in androgen receptors [63] and is affected by circulating androgens [64]—to fearful and angry faces co-varies positively with individual differences in serum testosterone concentrations [65, 66] (but see [67] for opposing results). In an experimental study, administration of testosterone was associated with increased amygdala reactivity to threat-related stimuli in young women [113]. These results are consistent with a longitudinal study in adolescents that found that increased levels of pubertal testosterone disrupted typical coupling between the amygdala and OFC, leading to increased amygdala reactivity to threat-related stimuli in both sexes [114]. Finally, whereas in boys higher testosterone levels are associated with lower activation in the striatum and PFC during processing of emotional conflict, in girls higher testosterone levels are associated with lower activation in the PCC/precuneus [115]. Other investigators have shown in both boys and girls that higher levels of testosterone are associated with higher striatal activation during reward consumption [116], and with higher striatal activation when adolescents select smaller yet more immediate rewards [117]. In an observational longitudinal neuroimaging study in male and female adolescents between the ages of 8–27 years, activation in the striatum (nucleus accumbens) to rewarding stimuli peaked during adolescence, and was associated with accompanying changes in testosterone levels [118]. In a recent study, in which salivary testosterone was measured in adolescents before and after a neuroimaging scan, acute increases in testosterone were associated with smaller differences in activation between reward cues signaling reward or non-reward outcomes for a given trial in vmPFC and posterior cingulate cortex (PCC) in both sexes [119]. Thus, by altering neural processing of both negative (e.g., threatening) and positive (e.g., rewarding) valenced stimuli, testosterone may be facilitating the expression of adolescent sensation-seeking and risk-taking behavior [118] through dopaminergic transmission among these subcortical and prefrontal circuits. Certainly, these processes may not be specific to STBs and may represent a more general diathesis for mental disorders that are characterized by sensation-seeking and risky behaviors; however, future research is needed to test this possibility more explicitly.

In sum, studies to date have demonstrated that higher levels of testosterone are associated with aspects of emotion-related impulsivity in males with a history of suicidal behaviors (e.g., attempts). Higher levels of testosterone are also associated with steeper temporal discounting of rewards driven by patterns of activation in striatal, vmPFC, and OFC in both sexes; sex differences in the effect of testosterone on adolescent brain circuits appear to be most prominent in emotion-related contexts. Indeed, in adults, both endogenenous levels [120] and exogenous administration [121] of testosterone enhance risk taking. The predisposition to take risky decision has been shown to be a critical risk factor for suicidal behavior in adults [122] and, to a lesser extent given the sparse literature on this topic, in adolescents [123]. Hence, functional alterations in decision-making systems induced by puberty-related rises in testosterone levels, or their context-dependent fluctuations, might be related to suicidal risk through an increase probability of perpetrating highly risky and self-destructive behaviors in the face of overwhelming stress (particularly social stressors involving loss of desired status). Future work is needed to test explicitly whether higher levels of testosterone exacerbate affect striatal and PFC systems underlying maladaptive responses to social stressors that, in turn, lead to heightened risk for STBs.

### DHEA

DHEA (and its sulfate, DHEA-S) is the most abundant steroid hormone in humans and is a precursor to sex steroids. Researchers have not yet examined the relation between DHEA (or DHEA-S) and STBs in adolescents; moreover, and the few studies that have been conducted with adults differ in their measurement of DHEA. One study showed that male suicide attempters had higher levels of DHEA-S than did healthy controls, and that exposure to extreme social threat (i.e., interpersonal violence) as a child was negatively correlated with the ratio of cortisol/DHEA-S [124]. Among adults diagnosed with PTSD, those who had attempted suicide had significantly higher levels of DHEA than did those without a history of attempt [125]. Another study found that, in combat veterans, the ratio of DHEA/DHEA-S was positively correlated with total adolescent aggression scores, total adulthood aggression scores, and lifetime aggression scores in those who had attempted suicide but not in nonattempters [126].

Overall, these studies suggest that higher levels of DHEA or DHEA-S may be associated with risk for STBs. Indeed, DHEA modulates neurotransmitter systems implicated in suicidal thoughts and mood disturbances, including acting as an antagonist for GABA receptors, with genes widely expressed in hippocampus, amygdala, and striatum [127]. There is also intriguing evidence that psychiatric conditions characterized by excessive stress and elevated suicide risk lead to a downregulation of neurosteroid biosynthesis—including the conversion of DHEA to GABAergic metabolites, such as allopregnanolone [128]—and changes in GABA_A_ receptor subunit composition [129]. Thus, STB risk may be associated with higher levels of DHEA due to an insufficient ability to metabolize DHEA. With respect to the effects of DHEA on the adolescent brain, however, there have been a small number of studies. Thus, far investigators have found that higher levels of DHEA are associated with larger hippocampal volumes in both male and female adolescents [130] and with lower white matter organization across a broad set of white matter tracts [110]. Other studies have found that higher DHEA levels are associated with reduced cingulate activation and greater functional connectivity between the amygdala with ACC and other regions involved in processing salient information in adolescents during the processing of social stimuli (e.g., viewing fearful faces) [131]. Moreover, higher DHEA levels are associated in girls with lower activation in cingulate regions implicated in processing salience information and with greater externalizing problems [132], but in boys with stronger functional connectivity between the amygdala and inferior frontal gyrus (the opposite pattern was found in girls) and with higher anxiety symptoms [131].

Despite the heterogeneity in clinical, developmental, and demographic features across the studies reviewed in this section, there appear to be consistent associations between higher levels of DHEA and both STBs and altered structure and function of brain circuits underlying emotion regulation. In adults with clinical disorders, higher levels of DHEA (either alone or relative to cortisol) appear to be associated with STBs. In psychiatrically healthy adolescents, higher levels of DHEA are associated with reduced downregulation of affective signals in the amygdala from emotion regulatory cortical regions, including the ACC and portions of the PFC. Thus, higher levels of DHEA may disrupt the development of emotion regulatory brain circuits across adolescence in ways that increase risk for STBs when individuals are exposed to interpersonal stressors (particularly those characterized by threat). However, it is worth noting one study of psychiatrically healthy adults in which administration of exogenous DHEA reduced activation in the amygdala and hippocampus, increased activation in the vmPFC, and led to stronger connectivity between the amygdala and hippocampus during an emotion reappraisal task; moreover, decreased activation in the hippocampus during the task was associated with lower negative affect, suggesting that higher levels of DHEA improve negative mood by downregulating affective signals in the hippocampus [133]. Additional research is needed to examine whether these associations hold in clinical samples of adolescents and whether (or how) they are related to risk for STBs.

## Consideration of sex differences

Across most countries, being female increases the risk of suicidal thoughts and related behaviors [5]. Despite this higher prevalence of STBs in women, men are more likely to die by suicide [134], leading to “the gender paradox of suicide.” It is notable that the sex difference in suicide deaths increases dramatically in adolescence [135], suggesting that puberty plays an important role in explaining this difference (although this sex difference is now narrowing among adolescents [136], indicating that other factors, including environmental or cultural influences, are also likely). Whereas past-year ideation, plans, and attempts tend to peak during mid-adolescence (~16 years) in girls, these rates increase steadily throughout mid- to late adolescence in boys [137, 138].

As in adults [5, 139], there are sex differences in suicide-related outcomes in adolescents that are mediated in part by differences in lethality and mental illness. In a psychological autopsy study, adolescent male suicide victims were more likely to use lethal methods and had a higher prevalence of conduct disorder than did female victims [140]. A recent meta-analysis of sex-specific suicide risk in adolescents (ages 12–26 years) found distinct clinical and environmental risk factors for suicide attempt in male and female adolescents [139]. Thus, in addition to the hormonal differences that underlie sex-specific neurodevelopmental changes, different clinical conditions (externalizing disorders in boys versus mood disorders in girls) and social stressors (peer influence in boys versus direct trauma/victimization from romantic relationships in girls) may further explain or moderate these pathways to risk for STBs. Because of the sparse literature, however, it is not clear whether sex-specific risk factors are present before puberty, whether puberty affects neuroendocrine systems in sex-specific ways to increase risk for STBs, or whether boys and girls are exposed to differential environmental risk factors as a result of going through puberty. Future research should attempt to clarify the extent to which pubertal processes play a central role in STBs or whether they are implicated in mental illness more generally.

While adrenal and gonadal hormones and physical maturation are important indicators of pubertal development, other aspects of puberty may be relevant in the context of adolescent risk for STBs. Pubertal timing—the age at which individuals mature relative to their peers [141]—has been linked to individual differences in mental disorders in adolescence [142, 143]. Moreover, the timing of pubertal onset may have a different impact on developing neuroendocrine function depending on whether it occurs earlier or later in a given individual [34, 139]. Given that girls typically enter puberty earlier than do boys, considering the role of pubertal timing may also elucidate sex differences in risk for STB—as well as mental illness more generally—and accompanying endocrine and neural processes. A growing number of studies are reporting that early menarche is associated with elevated risk for suicidal ideation in adolescent girls [144–146]. In a recent longitudinal study of a large birth cohort, earlier age of menarche was associated with increased suicidal behaviors at 16 and 21 years of age [147], suggesting that earlier puberty has an enduring effect on STB risk throughout adolescence and young adulthood. In addition, considerable evidence suggests that pubertal timing is influenced by early life adversity [148], which itself is a robust predictor of STBs [149]. Thus, early puberty resulting from early adversity may be a mechanism by which suicide risk is instantiated or exacerbated in vulnerable adolescents. Finally, it is important to acknowledge that sexual orientation and identification as a sexual minority are increasingly being recognized as risk factors for STBs [150]; more research in this area is critically needed.

## Future directions

No research has yet examined whether and how pubertal hormones affect neurodevelopmental trajectories of brain circuits that mediate social cognition and emotion-related impulsivity explain risk for STBs during adolescence. Studies are needed to clarify the role of pubertal timing and the multidimensional mechanisms—biological, social, cultural—by which puberty-related processes influence risk for STBs. An important next step for the field is to first establish reliable associations between pubertal hormones and adolescent brain structure and function and to then map those associations onto neural circuits underlying STBs in adolescents. Other critical knowledge gaps include disentangling neuroendocrine mechanisms that are more closely linked to suicidal thoughts versus attempts (and other self-harming behaviors) and that facilitate the transition from ideation to action [151].

Although we focused here on the effects of pubertal hormones, we should acknowledge that there is considerable evidence that HPA-axis dysfunction is associated with STBs and self-harming behaviors in adolescents [13, 21, 152, 153]. Puberty is also the time when HPG-axis regulation of the HPA axis develops [154–157]. Given that the expected suppression of the HPA axis by the HPG axis [158] is disrupted in individuals at risk for STBs (due to such factors altering developmental changes in stress response and regulatory systems, including experiences of adversity and exposure to trauma during early life [154]), it is critical that researchers explicitly examine the role of puberty in this context.

It is also important that researchers in this area consider moving away from a nomothetic framework for the prediction of STBs and instead adopting idiographic or person-centered models. Puberty is a highly individualized process; adolescents differ markedly in their hormone levels, their neurobiological sensitivity to typical endocrine changes [14], their pubertal timing, their neurodevelopmental trajectories, and their psychological responses to maturation. Certainly, measuring these variables at the individual level is challenging and will have to take into account population norms on several dimensions (e.g., ethnicity, sociodemographic factors). Absent such data, standardization within age bands or residualized scores (e.g., regressing pubertal stage on age to obtain a measurement of pubertal timing) can be used to capture individual variability (for a treatment of these issues, see [159]). Another solution is to use “dense sampling” approaches that leverage repeated assessments (e.g., self-reported mood states, saliva samples, neurobiological measurements) from the same individual in order to capture intra-individual variation in these processes [13], which we argue is critical for understanding the contribution of ovarian hormones in risk for STBs in adolescent females. Dense-sampling has the additional advantage of being able to identify proximal factors that lead to suicidal states and other related high-risk events, which are often transient and rarely captured in the laboratory or clinic [160].

Other important study design considerations include larger sample sizes of both sexes in order to detect sex differences more reliably. As we alluded to in the earlier sections of our review, there are other important confounds to consider that will affect the reliability and validity of estimating the effects of within-person changes in sex hormones on brain and behavior. These confounds include genetic factors, environmental influences, medical conditions, and cycle length (given that the range of menstrual cycles and periods of anovulation are longer in adolescents than in adults, particularly in the first few years post-menarche [161]). Finally, understanding the normative associations between changes in hormones with the structural and functional development of the brain is a critical next step that must be established before understanding how these alterations are altered in individuals at risk for STBs. Gaining a more comprehensive understanding of neurobiological trajectories and the mechanisms of neuroplasticity in adolescents as a consequence of puberty will also contribute to our knowledge of sex-specific psychiatric interventions and may help to address the heterogeneity of risk factors identified to date [5]. For instance, the granularity of data obtained from dense-sampling methods will facilitate the identification of individuals with hormonal sensitivity to the typical fluctuations occurring during puberty, as well as characterize within-person pubertal processes associated with responses to social stressors and poorer impulse control during heightened emotional conflict. With this information, researchers and clinicians will be able to stratify individuals on the basis of neuroendocrine risk (e.g., those with hormone sensitivity) and also to identify points in time (e.g., dramatic increases in stress perception or depressive symptoms, unusual variability in DHEA) during which an individual might benefit from immediate intervention.

## Summary and conclusions

**Fig. 2:**
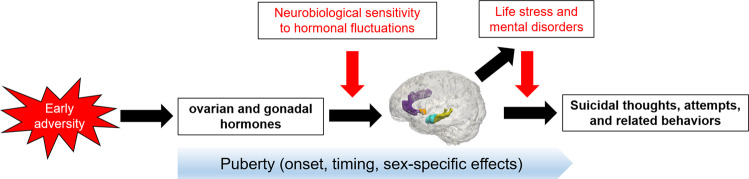
Conceptual model linking aspects of the pubertal transition with risk for suicidal thoughts and behaviors. Experiences of early adversity affect the programming and development of endocrine and neural systems which undergo significant maturation during puberty. Puberty-related changes in ovarian, gonadal, and other related hormones shape the neural circuits underlying social cognition, emotion regulation, and impulse control (which include structures such as the amygdala, hippocampus, striatum, anterior cingulate cortex, and portions of prefrontal cortex). Alterations in these circuits may partially explain the ways in which changes in sex hormones are linked with the emergence of suicidal thoughts and behaviors during adolescence. Moderators of these processes, including a neurobiological sensitivity to ovarian hormones, experience of ongoing life stressors, and underlying mental disorders, are highlighted in red.

Globally, suicide has surpassed all physical diseases as a cause of death in adolescents. Puberty drives psychobiological changes in adolescence that have not been examined explicitly in relation to suicide risk. In this review, we conceptualized suicidal thoughts and behaviors in adolescents as resulting from pathological responses to social stressors at a time when stress-regulatory systems are still maturing. We argued that alterations in brain circuits—comprised of connections among the amygdala, hippocampus, striatum, ACC, vmPFC, and OFC—that underlie social cognition, emotion regulation, and impulse control and are shaped by puberty-related changes in sex hormones. We propose that alterations in these circuits may partially explain the ways in which changes in sex hormones are linked with increased suicidal thoughts and behaviors during adolescence. However, we also highlighted critical moderators to be considered in this model, including a neurobiological sensitivity to fluctuations in ovarian hormones, exposure to early adversity, and underlying mental disorders (Fig. 2). To date, these specific hypotheses have not been tested. Although there is emerging research identifying shared and unique neural circuits that underlie suicidal thoughts and behaviors [68], it is paramount that that these associations be examined in prospective studies. It will also be important to use both experimental designs and large-scale longitudinal studies to elucidate the extent to which pubertal hormones affect the acute functioning of these circuits and drive the development of these circuits over the course of adolescence and young adulthood. Moreover, we expect that although pubertal hormones are not a primary driver of suicide risk, they may play an outsized role in individuals with a neurobiological sensitivity to hormonal fluctuations. Therefore, it is critical that we identify key moderators of the paths in our model, which we hypothesize includes neurobiological sensitivity to hormonal fluctuations, experiences of adversity and life stress that influence neurodevelopmental trajectories (which, in turn, may result in generating and experiencing additional stress), and underlying mental disorders. In addition to conducting longitudinal studies with larger sample sizes, we suggest that researchers also use dense-sampling methods to identify individuals according to these stratification parameters (e.g., hormonal sensitivity), as well as points in time at which individuals may be at risk and could benefit from more immediate intervention. In conclusion, we want to emphasize that increasing our understanding of pubertal science across endocrine, neural, and psychosocial domains will yield significant insights concerning how best to reduce the frequency of suicide-related deaths during the vulnerable period of adolescence.
